# Speech-Induced Suppression for Delayed Auditory Feedback in Adults Who Do and Do Not Stutter

**DOI:** 10.3389/fnhum.2020.00150

**Published:** 2020-04-24

**Authors:** Akira Toyomura, Daiki Miyashiro, Shinya Kuriki, Paul F. Sowman

**Affiliations:** ^1^Graduate School of Health Sciences, Gunma University, Maebashi, Japan; ^2^Faculty of Medicine, School of Health Sciences, Gunma University, Maebashi, Japan; ^3^Gunma University Hospital, Maebashi, Japan; ^4^Faculty of Health Sciences, Hokkaido University, Hokkaido, Japan; ^5^Department of Cognitive Science, Macquarie University, Sydney, NSW, Australia; ^6^Perception and Action Research Centre, Faculty of Human Sciences, Macquarie University, Sydney, NSW, Australia

**Keywords:** speech-induced suppression, delayed auditory feedback, auditory evoked potentials, stuttering, EEG

## Abstract

Speech-induced suppression is the normal, relative amplitude reduction of the auditory evoked potential for self-, compared to externally-generated, auditory stimulation. It remains controversial as to whether adults who stutter exhibit expected auditory modulation during speech; some studies have reported a significant difference between stuttering and fluent groups in speech-induced suppression during speech movement planning, while others have not. We compared auditory evoked potentials (N1 component) for auditory feedback arising from one’s own voice (Speaking condition) with passive listening to a recording of one’s own voice (Listening condition) in 24 normally-fluent speakers and 16 adults who stutter under various delayed auditory feedback (DAF) time conditions (100 ms, 200 ms, 500 ms, and 1,000 ms). We presented the participant’s own voice with a delay, immediately after presenting it without a delay. Our working hypothesis was that the shorter the delay time, the more likely the delayed sound is perceived as self-generated. Therefore, shorter delay time conditions are proposed to result in relatively enhanced suppression of the auditory system. Results showed that in fluent speakers, the shorter the delay time, the more the auditory evoked potential in the Speaking condition tended to be suppressed. In the Listening condition, there was a larger evoked potential with shorter delay times. As a result, speech-induced suppression was only significant at the short delay time conditions of 100 and 200 ms. Adults who stutter did not show the opposing changes in the Speaking and Listening conditions seen in the fluent group. Although the evoked potential in the Listening condition tended to decrease as the delay time increased, that in the Speaking condition did not show a distinct trend, and there was a significant suppression only at 200 ms delay. For the 200 ms delay condition, speakers with more severe stuttering showed significantly greater speech-induced suppression than those with less severe stuttering. This preliminary study suggests our methods for investigating evoked potentials by presenting own voice with a delay may provide a clue as to the nature of auditory modulation in stuttering.

## Introduction

Stuttering is a fluency disorder that prevents smooth production of speech. Repetitions (co-co-co-coffee), prolongations (cooooooffee), and blocks (…… cooffee) are the core symptoms characterizing the dysfluencies of stuttering. The population incidence ranges from 1 to 11% (Craig et al., 2002; McLeod and Harrison, 2009; Boyle et al., 2011; Reilly et al., 2013), and 60–80% of the cases of developmental stuttering recover without intervention (Kefalianos et al., 2017; Shimada et al., 2018). However, the remainder will often continue to experience lifelong speech dysfluency. Although numerous studies have reported potential neurobiological mechanisms underlying stuttering, at present no definite cause nor reliable treatment that all researchers accept, exists. Considering that the prevalence of stuttering is not small (around 1%; Yairi and Ambrose, 2013) and persistent stuttering often has a long-term negative impact on quality of life (Craig and Tran, 2014; Smith et al., 2014), investigating and describing the mechanisms and nature of stuttering remain an important endeavor.

There exist conditions under which stuttering can be transiently alleviated; both synchronization of speech with another person (the chorus effect; Andrews et al., 1982) and auditory feedback transformations, where the voice is pitch-shifted and/or time-delayed (Lincoln et al., 2006), are conditions under which dysfluency is temporarily suppressed. In general, distinguishing between externally produced sounds and those which are self-produced by one’s own speech is a function important for speech-related behaviors. Sensory representations of sounds are used to monitor for salient, action-triggering signals in the external environment, whereas self-produced vocal sounds are important inputs into auditory feedback pathways necessary for control of the speaker’s own vocal production. Making the distinction between sensory experiences created by one’s self vs. another is therefore an important auditory processing function. Moreover, it is not a process limited to hearing, but one common to all sensory domains. The distinction of self-produced afference from that produced by an external source gives rise to a number of interesting behavioral phenomena, e.g., self-produced tactile stimulation does not tickle, whereas that produced by another might (Blakemore et al., 1998). To account for such differences in sensory experience, the concept of an internal forward model has been proposed (Wolpert et al., 1995). In the auditory domain, an efference copy (a copy of the speech motor command), also known as a corollary discharge (Sperry, 1950; Crapse and Sommer, 2008), is sent to the auditory cortex in parallel with the motor command for the speech sent to the motor cortex. This “forward” prediction of the auditory consequence of one’s own speech, results in relative suppression of the auditory cortex response to one’s own voice, compared to that in response to an externally generated sound, i.e., speech-induced suppression (Numminen et al., 1999; Curio et al., 2000; Houde et al., 2002; Martikainen et al., 2005; Christoffels et al., 2011).

Speech-induced suppression in people who stutter has been examined in some previous studies. Daliri and Max (2015b) recorded the event-related potential in response to a probe tone (1-kHz pure tone) during speech movement planning. They reported that although fluent speakers showed a statistically significant modulation of the auditory evoked potential (reduced N1 amplitude), adults who stuttered did not show any significant modulation. They speculated that stuttering is associated with deficiencies in modulating the cortical auditory system during speech movement planning. This conclusion was followed-up later by Daliri and Max (2015a) who suggested that general auditory prediction difficulties exist in adults who stutter. However, similar studies from other laboratories [magnetoencephalography (MEG) studies: Beal et al., 2010, 2011; electroencephalography (EEG) study: Liotti et al., 2010], did not find a significant difference between stuttering and fluent groups. The major methodological difference between studies by Daliri and Max (2015a,b, 2018), and studies from other groups (Beal et al., 2010, 2011; Liotti et al., 2010) is that to measure auditory evoked potentials, the former studies presented pure tones during speech movement planning, while the latter studies used the speakers’ own voice. Therefore, these conflicting findings indicate that the atypical modulation of the auditory system in adults who stutter may not be induced when they perceive their own voice as an auditory stimulus, but instead may only be induced when perceiving sound stimuli other than their own voice, such as pure tones. In a recent study by Daliri and Max (2018), deficient auditory modulation (reduced speech-induced suppression) in adults who stutter normalized (increased) when they spoke under a delayed auditory feedback (DAF) condition, while that of fluent speakers decreased. Although Daliri and Max (2018) examined auditory modulation under DAF conditions, they only investigated the effect of a 100-ms delay condition. Also, considering that auditory attention differs when hearing pure tones and one’s own voice and that the N1 component is modulated by selective attention (e.g., Coles et al., 1990), it is not clear whether adults who stutter still show atypical modulation of the auditory system when they perceive their own voice at various DAF times.

In the current study, we compared auditory evoked potentials for auditory feedback arising from one’s own voice (Speaking condition) with that for passive listening to a recording of one’s own voice (Listening condition) under various DAF time conditions (100 ms, 200 ms, 500 ms, and 1,000 ms). To test the effect of hearing one’s own voice under DAF conditions, we presented the participant’s own voice again with a delay immediately after presenting auditory feedback without a delay. This experimental paradigm may be able to infer how much the auditory system is suppressed by efference copy when vocalizing under DAF conditions. The shorter the delay time, the more likely the delayed sound is perceived by participants as their own voice generated by themselves. Consequently, shorter delay time conditions are considered to induce stronger suppression of the auditory system. We examined the cortical activity in stuttering and fluent speakers by using our experimental methods and inferred the possibility of a deficiency in modulating the cortical auditory system during speech production.

## Materials and Methods

### Participants

Twenty-four fluent speakers (12 women, mean age = 19.8, SD = 2.0) and 16 adults who stutter (three women, mean age = 27.7, SD = 6.8) participated. None of them reported a history of speech, language, or hearing problems. All participants were native Japanese speakers. Three fluent speakers were left-handed and the others were right-handed, as assessed by the Edinburgh Handedness Inventory (Oldfield, 1971). Among the stuttering group, one was left-handed and the others were right-handed. There was a significant difference in age between groups (*t*_(16.7)_ = 4.39, *p* < 0.01). Therefore, we applied the analysis of covariance (ANCOVA) where age was a covariate if the ERP data satisfied the assumption that underlies the use of ANCOVA (see “Analysis section”). This study and protocol were approved by the Gunma University Ethical Review Board for Medical Research Involving Human Subjects. Written informed consent was obtained from all individuals before they participated following the Declaration of Helsinki.

Before the experiment, stuttering participants engaged in a conversation session in front of the experimenter and their speech was video-recorded. The severity of their stuttering was evaluated as percent syllables stuttered (%SS) based on video-recorded speech samples. We counted the core behaviors of stuttering in speaking, including repetitions, prolongations, blocking, and interjections due to blocking. The %SS ranged from 0.14% to 8.92% (mean = 1.96, SD = 2.31). Although this study included adults with very mild stuttering severity (e.g., 0.14%SS), such speakers disclosed that they generally stuttered more in more difficult situations and so they were included in the sample. To determine the measurement reliability of the evaluation, a second evaluator also independently identified stuttering episodes on the videos for four of the participants who stuttered (25% of the data; Guitar, 2005). Point-by point agreement was 97.5% on average, which was calculated as the number of agreements between two raters divided by the total number of agreements plus disagreements.

## Experimental Paradigm

### EEG Setups

The methods of subsequent EEG experiments were the same in both groups. The experiment was conducted within a shielded room. EEG was recorded from silver-silver chloride electrodes placed at Fz, Cz, Pz, C3, C4, T3, and T4 according to the international 10–20 system with a digital amplifier (Neurofax EEG 1200, Nihon Kohden, Tokyo, Japan). All electrodes were referenced to the average of the two earlobes. A ground electrode was placed on the forehead (Fpz). To monitor blinks, electrooculograms (EOGs) were recorded *via* electrodes placed above the left eye and below the right eye. All signals were digitized at a sampling rate of 1,000 Hz. The impedance of all electrodes was kept below 5 kΩ. Participants were required to perform the following two kinds of tasks (Speaking task and Listening task) under 100 ms, 200 ms, 500 ms, and 1,000 ms DAF.

### Speaking Task

In this study, participants were instructed to speak /a/ very lightly, without moving their mouths very much and with their mouth slightly opened; it is not feasible to investigate EEG signals during continuous speech because speech-related electromyogram artifacts negatively affect EEG signals. However, in pilot experiments, we visually confirmed that our method, where participants were instructed to speak very lightly, without moving their mouth very much and with their mouth slightly open, did not induce significant EEG artifacts. Using this speaking method, participants were required to vocalize /a/ 100 times per condition during EEG acquisition. Participants repeatedly practiced this speaking method while they were monitoring a VU meter (AMU-2SII, TOMOKA, Tokyo, Japan) before the experiment. The sound pressure of the vocalization was about 77 dB SPL (LAFmax), which was measured at 10 cm from the mouth at an angle of 30 degrees *via* a sound level meter (Type 2250, Brüel and Kjær, Naerum, Denmark). Participants were told to minimize blinking as much as possible. DAF behavioral experiments often incorporate pink noise to suppress the effects of bone conduction. However, it is known that noise-masking differentially affects the EEG signals of people who stutter compared to fluent speakers (Saltuklaroglu et al., 2017). Therefore, we did not use pink noise masking in this experiment.

**Figure 1:**
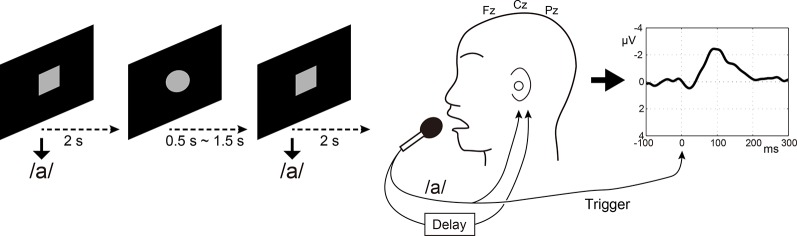
Speaking condition in the electroencephalography (EEG) experiment. Participants were instructed to vocalize /a/ very lightly soon after that a gray circle drawn on a black background changed to a gray square. Three midline electrodes Fz, Cz, and Pz were used for calculating auditory evoked potential in response to auditory feedback of speech. The speech signal was sent into an auxiliary EEG channel for offline-extraction of the onsets of individual speech.

Participants’ voices were recorded through a microphone (SM58, SHURE, Niles, IL, USA) at a distance of 3 cm from the participants’ mouth. Speech was fed back to the participant *via* insert earphones (ER4 microPro, Etymotic Research, Elk Grove Village, IL, USA) through an artificial auditory feedback circuit. Simultaneously, the speech was sent to a delay circuit incorporated in an effects unit (Eclipse, Eventide, Little Ferry, NJ, USA) to realize the DAF condition. This was fed back to participants’ ears with a delay at the same sound pressure level (Figure 1). The speech signal was sent into an auxiliary EEG channel for offline-extraction of onsets of individual speech. Because the sampling rate (1,000 Hz) was low for recording voice signal, the voice was also sent to another PC and was recorded with Audition CS6 (Adobe Systems, San José, CA, USA) at a sampling rate of 44.1 kHz. The recorded voice sampling at 44.1 kHz was also used in the subsequent Listening task. Timing of speech was instructed by visual stimuli implemented using Psychtoolbox-3^1^ running on MATLAB (MathWorks, Inc., Natick, MA, USA): participants were instructed to vocalize soon after a gray circle is drawn on a black background changed to a gray square (Figure 1). To prevent anticipatory or rhythmic speaking on the part of the participants, the onsets of successive speech cues were temporally jittered: the gray circles were presented for 0.5–1.5 s, and gray squares for 2 s immediately after the circles, Therefore, the participants vocalized /a/ every 2.5–3.5 s. One run for each condition (100 ms, 200 ms, 500 ms, and 1,000 ms delay time conditions) lasted about 5 min. The order of delay time was randomly assigned between participants. Because the long-latency auditory evoked potential (N1 component) is observed at around 100 ms post-stimulus, we set the minimum delay time to be 100 ms so that the N1 component for the next sound was not mixed with the N1 for the first sound.

### Listening Task

Following the Speaking condition at each delay time (e.g., 100 ms delay), the Listening task with the same delay condition (e.g., 100 ms delay) was conducted. Participants were required to passively listen to their own voice which was recorded at a sampling rate of 44.1 kHz with Audition CS6 during the Speaking task. A black circle on a gray background was presented as an eye fixation point during this session. Similarly to the Speaking task, the voice signal was also sent to a delay circuit. Therefore, the series of sounds presented was the same as that for the Speaking condition and included both the directly vocalized sound and the delayed sound. The sound pressure of the stimuli was the same as that for the Speaking condition. The voice signal was sent into an auxiliary EEG channel for offline-extraction of the onsets of individual sound stimuli.

## Analysis

### Voice Onset Extraction

Voice onsets, from the speech waveforms recorded on the auxiliary EEG channel and Audition CS6, were extracted in MATLAB for the calculation of auditory evoked potentials in response to auditory feedback of voice. The waveform was Hilbert transformed and the amplitude envelope calculated. The speech onset matrix was created by regarding the case where the envelope of the waveform was above a threshold. The threshold was visually determined for each participant. Finally, extracted onset timings and waveforms were overlapped and the onset matrix corrected manually.

### Auditory Evoked Potentials

EEG data were analyzed in BrainVision Analyzer2 (Brain Products, Gilching, Germany). Independent component analysis (ICA) correction was applied to remove artifacts due to EOG activity. An IIR bandpass filter (0.1–30 Hz) was applied to all data sets to minimize the effect of high-frequency noise sources such as powerline interference or electromyographic activity, as well as low-frequency slow voltage changes (Luck, 2014). The baseline for auditory evoked potential segmentation was defined as −100 ms to 0 ms before voice onset. Automatic artifact rejection was applied to remove epochs containing large drifts. Also, epochs containing artifacts were eliminated by visual inspection for all segments. Artifact-free epochs were averaged to compute auditory evoked potentials. Three midline electrodes Fz, Cz, and Pz were used for calculating auditory evoked potentials in response to auditory feedback of speech. The N1 component was automatically inspected within the window of 50–150 ms post-speech onset. The analysis methods used here are largely the same as those used in our previous study (Miyashiro et al., 2019).

In the current experiment, we calculated evoked potentials locked to the speech onset time rather than the feedback onset time. This design was employed for the following reason: The efference copy is sent at the moment the speaker produces speech, and speech-induced suppression is time-locked to this event. If we were to evaluate speech-induced suppression of the delayed feedback signals (i.e., calculate evoked potentials for DAF of voice between 100 ms and 1,000 ms), we would not expect to observe significant suppression as the suppression epoch would likely have passed already—especially at long delays. However, by requiring participants to vocalize /a/ 100 times under the same delay-time condition, the participants could predict the delayed sound at the timing of vocalization. Therefore, even if we calculated the speech-induced suppression locked to the speech onset, we hypothesized that behaviorally effective DAF time (i.e., around 200 ms), which is a peculiar delay that confuses the speakers, would differentially affect the speech-induced suppression.

Participants who showed noisy EEG data or who did not show clear N1 peak in the Listening condition were excluded from the analysis. Accordingly, eight participants from the 24 members of the fluent speaker group, and four from the 16 members of the stuttering group were excluded from the analysis.

ANCOVA was performed treating the participants’ age as a covariate. First, we assessed the following assumption that underlies the use of ANCOVA, the dependent variable increases or decreases as the covariate increases or decreases. Alternatively, a significant correlation is assumed between the covariate and the dependent variable. The N1 amplitude did not significantly correlate with the covariate (age) for any delay condition in either speaker group in our sample (*p* > 0.05). This non-significant effect does not satisfy the assumption that underlies the use of ANCOVA. Therefore, we performed an analysis of variance (ANOVA). A three-way ANOVA of N1 amplitude with the factors of group (fluent vs. stuttering group), task (Listening vs. Speaking conditions), and delay time (100 ms, 200 ms, 500 ms, and 1,000 ms) was performed. Also, we performed a two-way ANOVA with factors of group and delay time on speech-induced suppression (Listening—Speaking). Based on our *a priori* hypothesis, multiple comparisons using Tukey’s HSD test were performed between the Listening and Speaking conditions in each group and for each delay time (100 ms, 200 ms, 500 ms, and 1,000 ms) to investigate whether speech-induced suppression was significant. The relationship between the stuttering frequency (% SS) of each speaker in the stuttering group and the magnitude of the N1 amplitude of each condition was investigated by Spearman’s rank correlation analysis.

To investigate the change in N1 amplitude due to the increase in delay time, a regression analysis was performed for each participant using the four delay times as independent variables and N1 amplitude as a dependent variable, and regression coefficients were calculated. Using a one-sample *t*-test we investigated whether the calculated regression coefficients were significantly different from zero. Furthermore, a two-way ANOVA of the regression coefficient, with the factors of group and condition, was conducted to investigate the effect of each on the regression coefficient.

## Results

**Figure 2:**
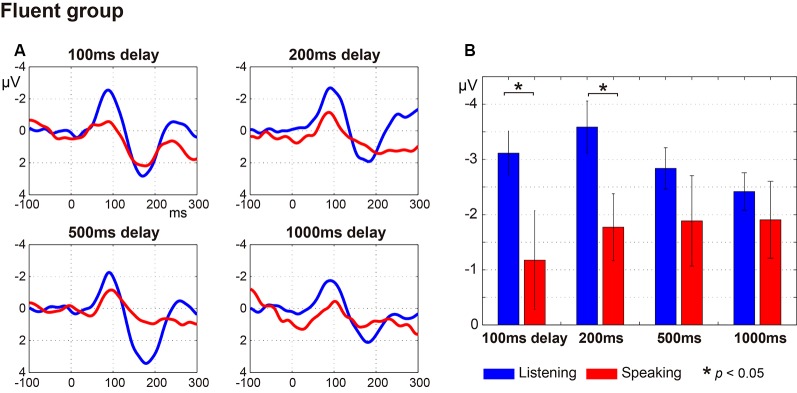
Auditory evoked potentials in fluent speakers. **(A)** Averaged auditory evoked potentials under each delay time condition. The blue line represents the Listening task and the red line represents the Speaking task. **(B)** N1 amplitude extracted from each participant’s auditory evoked potentials. Graphs represent mean ± SEM. There were significant differences between Listening and Speaking conditions for the N1 amplitude under the 100 ms and under 200 ms (*p* < 0.05) delay conditions with Tukey’s HSD test, but no significant differences under the 500 ms or 1,000 ms delay conditions.

**Figure 3:**
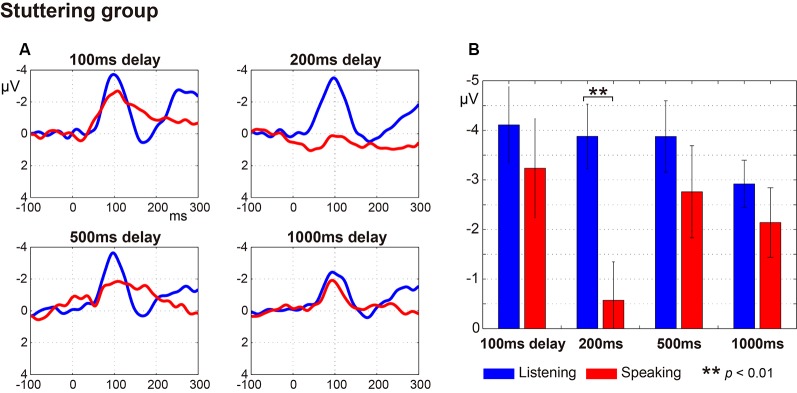
Auditory evoked potentials in the stuttering group. **(A)** Averaged auditory evoked potential under each delayed auditory feedback (DAF) time condition. The blue line represents the Listening task and the red line represents the Speaking task. **(B)** N1 amplitude extracted from each participant’s auditory evoked potential. Graphs represent mean ± SEM. Only the 200 ms delay condition showed a significant difference in comparison with Tukey’s HSD test between the N1 amplitude for Listening and Speaking conditions (*p* < 0.01).

Figure 2 (fluent group) and Figure 3 (stuttering group) display: (a) the ERP waveforms; and (b) the amplitude of the N1 component at a latency of around 100 ms (window of 50–150 ms post-speech onset) under the four delay conditions. In the fluent group, averaged ERP waveforms show clear speech-induced suppression (Listening > Speaking) for all conditions (Figure 2). In the stuttering group, by contrast, although the waveforms for the 100 ms, 200 ms and 500 ms delay conditions show speech-induced suppression, the waveforms for the 1,000 ms delay condition did not (Figure 3).

A three-way ANOVA with factors of group, condition, and delay time on N1 amplitude showed that there was a significant main effect only for condition (Listening vs. Speaking; *F*_(1,26)_ = 15.46, *p* < 0.001), demonstrating that speech-induced suppression was evident in this experiment. There were no significant effects or interactions for group or delay time. Also, a two-way ANOVA with factors of group and delay time on speech-induced suppression (Listening—Speaking) did not show significant effects or interactions for group or delay time.

Multiple comparisons were conducted using Tukey’s HSD test between Listening and Speaking conditions based on* a priori* hypotheses. In the fluent group, there were significant differences between Listening and Speaking conditions for the N1 amplitude under the 100 ms and 200 ms delay conditions (*p* < 0.05), but no significant differences under the 500 ms or 1,000 ms delay conditions. In the stuttering group, a significant difference between Listening and Speaking conditions only occurred for the 200 ms delay condition (*p* < 0.01). The 100 ms, 500 ms, and 1,000 ms delay condition did not yield significant effects. These results showed that, in both groups, only the short delay time conditions (100 ms and/or 200 ms) induced significant suppression of the Speaking condition compared to the Listening condition.

**Figure 4:**
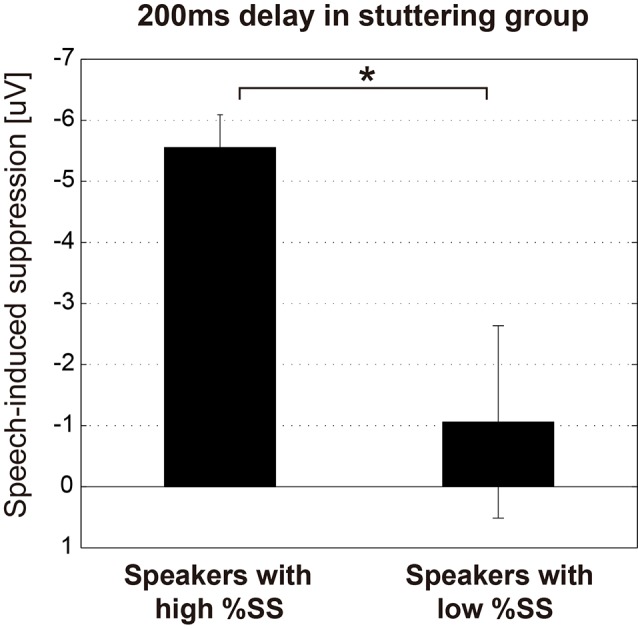
Magnitude of the speech-induced suppression under the 200 ms condition of the stuttering group. The stuttering group (*n* = 12) was divided into two subgroups (*n* = 6 vs. 6) by the median value of %SS. Graphs represent mean ± SEM. There was a significant difference between these subgroups (*t*_(10)_ = 2.702, *p* < 0.05), suggesting that more severely stuttered speakers suppressed the perception of their auditory feedback voice more when vocalizing /a/ under the 200 ms DAF condition. Star (*) indicates *p* < 0.05.

The relationships between the stuttering frequency (%SS) of each speaker in the stuttering group, and the magnitude of the N1 amplitude of each condition (Listening and Speaking conditions) as well as the speech-induced suppression (Listening—Speaking), were examined by Spearman’s rank correlation analysis. Through this analysis, we found that the auditory evoked potential was significantly modulated by stuttering frequency only for the 200 ms delay condition, where significant speech-induced suppression was found. For the 200 ms delay condition, the N1 amplitude in the Speaking condition (*r* = 0.580, *p* < 0.05) and the magnitude of speech-induced suppression (*r* = –0.636, *p* < 0.05) were significantly correlated with %SS, but the N1 amplitude in the Listening condition (*r* = –441, *p* = 0.15) was not. In all other conditions (Speaking, Listening conditions and the speech-induced suppression, under 100, 500, 1,000 ms delay conditions), amplitudes did not significantly correlate with %SS. We divided the stuttering group (*n* = 12) into two subgroups (*n* = 6 vs. 6) by the median %SS, and compared the speech-induced suppression (Listening—Speaking) for the 200 ms condition (Figure 4). Speakers with more severe stuttering showed significantly greater speech-induced suppression than speakers with less severe stuttering (*t*_(10)_ = 2.702, *p* < 0.05). This result indicates that, when vocalizing /a/ under the 200 ms DAF condition, speakers with more severe stuttering suppressed the perception of their auditory feedback more. Participants with relatively severe stuttering among the participants thus contributed most to the significant speech-induced suppression in the 200 ms delay.

**Figure 5:**
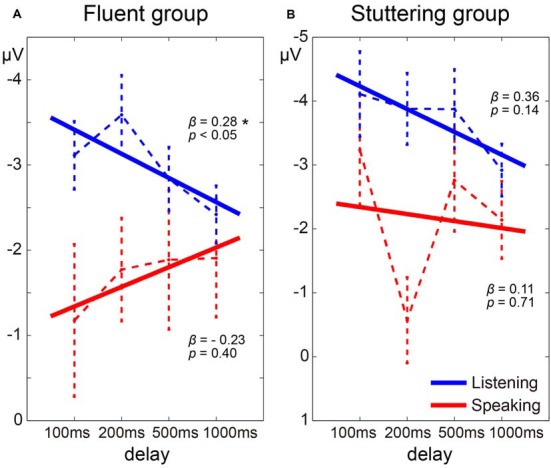
Delay time dependence of auditory evoked potentials in the fluent group **(A)** and stuttering group **(B)**. The dotted line represents the transition of the average of the evoked potential under each delay time condition and the solid line represents the average of the regression lines estimated from each participant. Note that because the Y-axis is inverted, the beta value is opposite in sign to the slope of the regression line. Star (*) indicates *p* < 0.05.

Regression coefficients were estimated for each task (Speaking and Listening) in each participant. In the fluent group, as the delay time increased, the N1 amplitude in the Listening condition tended to decrease, while the N1 amplitude in the Speaking condition tended to increase (Figure 5A). The mean regression coefficient (*β*) in the Listening condition in this group was 0.28 and was significantly different from zero (*t*_(15)_ = 2.14, *p* < 0.05). The coefficient in the Speaking condition in the same group was –0.23, but this was not significantly different from zero (*t*_(15)_ = 0.86, *p* = 0.40). In the stuttering group, however, although the N1 amplitude in the Listening condition tended to decrease as the delay time increased, a consistent trend was not noted in the Speaking condition (Figure 5B). The mean regression coefficient in the Listening condition in the stuttering group was 0.36 and was not significantly different from zero (*t*_(11)_ = 1.60, *p* = 0.14). The coefficient in the Speaking condition in this group was 0.11 and not significantly different from zero (*t*_(11)_ = 0.39, *p* = 0.71). A two-way ANOVA of the regression coefficient with the factors of group and condition did not reveal a significant effect of group, condition, or an interaction.

## Discussion

The results of the current study show that for fluent speakers, auditory evoked potentials in the Listening condition significantly decreased as the DAF delay time increased (from 100 ms to 1,000 ms). Evoked potentials in the Speaking condition tended to increase as the delay time increased. A novel aspect of this study is that we presented the participant’s own voice again with a delay (100 ms, 200 ms, 500 ms, or 1,000 ms) immediately after presenting auditory feedback sound without a delay. We interpret our findings to mean that the shorter the delay time, the more likely that feedback is perceived as one’s own voice. In the longer delay times, such as 500 ms or 1,000 ms, although the delayed sound could be “recognized” as their own voice, the sound might not be perceived as the voice that they just generated by themselves. Analogously, the rubber hand illusion persists with delays between visual and tactile feedback up to approximately 300 ms but decays at longer delays, i.e., the recognition of feedback as being self-induced has been demonstrated to be robust to short delays in other modalities (Shimada et al., 2009). In the present experiment, the shorter the delay time, the more the auditory system was suppressed by efference copy. Feedback evoked relatively small potentials for the short delay conditions (100 ms and 200 ms) in the Speaking condition. However, in the Listening condition, where the recorded sound is presented passively, the shorter the delay, the higher the sound density per unit of time (i.e., two successive sounds with a 100-ms interval concentrate more energy in a short time than two successive sounds with a 1,000-ms interval). This, in turn, causes a larger amplitude auditory evoked potential.

As a result of these opposite trends between Speaking and Listening conditions, speech-induced suppression decreased as the delay time increased, and significant suppression was observed only with short delay times (100 and 200 ms) in the fluent group. For normally fluent speakers, speech production under DAF conditions is a state where confusion occurs due to mismatches between auditory feedback of voice and its prediction. Therefore, this result also could be interpreted as being an attempt to avoid the confusion caused by DAF, by suppressing the perception of the auditory feedback sound that induces the confusion. However, the question remains as to why it only happens with short delays. The speech used in this experiment was not continuous speech but rather a short vocalization of /a/, thus we cannot directly compare the present study with experiments using continuous speech tasks. However, significant suppression at short delay times is consistent with the findings of traditional DAF studies where short delay times are most effective in disturbing continuous speech production (e.g., Lee, 1950; Black, 1951; Fairbanks, 1955; Yates, 1963; Kalinowski et al., 1993; Lincoln et al., 2006). Further studies incorporating continuous speech tasks are necessary to clarify the mechanism of auditory suppression at short delay times.

The stuttering group also showed a tendency for decreased evoked potentials as the delay time increased in the Listening condition. However, in the Speaking condition, a consistent trend, such as that seen in the fluent group, was not evident. A significant suppression was noted only in the 200-ms delay condition. Also, the slope of the relationship between evoked potentials and the delay tended to decrease rather than increase as was the case in the fluent group, though a statistically significant difference in the regression coefficients between groups was not detected.

Both groups showed speech-induced suppression with 200 ms DAF, suggesting that 200 ms is critical in the auditory feedback loop regardless of the speaker. Subgroup analysis within the stuttering group indicated that speakers with more severe stuttering contributed most to the significant speech-induced suppression at the 200 ms delay (Figure 4). Speakers with severe stuttering are more likely to cope with a stuttered speech in conversation by paraphrasing and choosing words, due to their frequent disfluency. We speculate that at the critical delay time condition (200 ms), participants with more severe stuttering might try to adapt to the DAF condition, which is a state that induces confusion, by suppressing the perception of auditory feedback voice even in a simple vocalization task. A similar result was found in a MEG study on children who stutter; Beal et al. (2011) reported that children who stutter with more severe stuttering showed lower left hemisphere M50 amplitude in the auditory cortex when vocalizing /a/. However, another study on adults who stutter by the same group did not find a significant correlation (Beal et al., 2010).

Our result of no significant group difference in the magnitude of speech-induced suppression (Listening vs. Speaking) is not consistent with the results of a series of works by Daliri and Max (2015a,b, 2018) but do agree with Beal et al. (2010) and Liotti et al. (2010), neither of whom found group differences in speech-induced suppression. These apparent discrepancies should be considered in the context of important methodological differences in the studies mentioned; Daliri and Max (2015a,b, 2018) presented a pure tone to participants whereas Beal et al. (2010) and Liotti et al. (2010), along with our experiment presented participants’ own voice as an auditory stimulus. Furthermore, the timing of presenting the stimuli were different; the studies by Daliri and Max (2015a,b, 2018) presented the auditory stimulus during speech movement planning, whereas Beal et al. (2010) and Liotti et al. (2010), and our experiment presented the auditory stimulus during speech production (immediately after speech onset). It is therefore difficult to derive a coherent conclusion from these results as a whole, though at a minimum there is consistent evidence that the magnitude of speech-induced suppression when speakers listen to their own voice through auditory feedback during speech production is likely not to differ between adults who do and do not stutter. Another study that used both pure tone and speech sounds (first consonant-vowel of a word) presented during speech movement planning reported that the amplitude of N1 was comparable between groups, but the latency of P200 was longer in adults who stutter than in fluent speakers (Mock et al., 2015). The stuttering of participants in this study was mild (mean %SS was 1.96), which also may be a reason for not finding a significant difference between groups.

Several neuroimaging studies (functional MRI and PET) have reported that adults who stutter showed lower auditory cortex activity than fluent controls when they speak (Fox et al., 1996; Brown et al., 2005; De Nil et al., 2008; Budde et al., 2014; Toyomura et al., 2015). The speech conditions used in these neuroimaging studies induce longer sound stimuli (auditory feedback sound) than our experiment. Therefore, although we cannot directly compare the studies of evoked potentials (evoked fields) with these neuroimaging studies, the finding of lower auditory cortex activity reported in neuroimaging studies is not consistent with our results (evoked potential in Speaking condition was not different between groups) nor those of Daliri and colleagues (stuttering speakers fail to suppress the auditory cortex; Daliri and Max, 2015a,b, 2018).

Because the experimental design of this study was novel, rather than replicating previous studies, there is a necessity for follow-up studies. We did not measure a non-DAF condition. The presence or absence of the lack of auditory modulation in adults who stutter could be considered in more detail by comparing the auditory evoked potentials in DAF with non-DAF conditions. Also, we focused on the amplitude of the evoked potential and did not measure latencies. The inclusion of the evaluation of latency would highlight another aspect of the auditory cortex’s response to speaking. As discussed above, the fact that the stuttering of participants in this experiment was relatively mild might have led to the non-significant difference between groups. We also did not systematically collect treatment history from stuttering participants in this study—another variable that might bear upon the findings.

In summary, this preliminary study showed that, in fluent speakers, the auditory evoked potential in response to feedback with one’s own voice increased as the delay time increased, but the pattern reversed when listening to a recorded voice. Adults who stutter did not show a clear trend when speaking in delayed feedback conditions. However, speech-induced suppression was most evident for short delay times (100–200 ms) in both groups. Because of the limitations of our study design, further studies are required to reach a definitive conclusion regarding whether stuttering is associated with atypical speech-induced suppression during the speech.

## Data Availability Statement

The datasets supporting the conclusions of this article are available on reasonable request to the corresponding author.

## Ethics Statement

This study and protocol were approved by the Gunma University Ethical Review Board for Medical Research Involving Human Subjects. Written informed consent was obtained from all individuals prior to their participation in accordance with the Declaration of Helsinki.

## Author Contributions

AT designed the study and analyzed the data. AT and SK discussed the preliminary studies. AT and DM conducted the experiment. AT, SK, and PS discussed the results. AT and PS wrote the article.

## Conflict of Interest

The authors declare that the research was conducted in the absence of any commercial or financial relationships that could be construed as a potential conflict of interest.

## Footnotes

^1^http://psychtoolbox.org/
